# Leveraging the trend analysis for modeling of the greenhouse gas emissions associated with coal combustion

**DOI:** 10.1007/s11356-024-34654-3

**Published:** 2024-08-16

**Authors:** Izzet Karakurt, Busra Demir Avci, Gokhan Aydin

**Affiliations:** Mining&Energy Research Group, Mining Engineering Department, Karadeniz Technical University, Ortahisar, 61080 Trabzon, Turkey

**Keywords:** Trend analysis, Modeling, Greenhouse gas emissions, Coal combustion

## Abstract

In this paper, it is aimed, for the first time, at deriving simple models, leveraging the trend analysis in order to estimate the future greenhouse gas emissions associated with coal combustion. Due to the expectations of becoming the center of global economic development in the future, BRICS-T (Brazil, the Russian Federation, India, China, South Africa, and Turkiye) countries are adopted as cases in the study. Following the models’ derivation, their statistical validations and estimating accuracies are also tested through various metrics. In addition, the future greenhouse gas emissions associated with coal combustion are estimated by the derived models. The results demonstrate that the derived models can be successfully used as a tool for estimating the greenhouse gas emissions associated with coal combustions with accuracy ranges from at least 90% to almost 98%. Moreover, the estimating results show that the total amount of greenhouse gas emissions associated with coal combustions in the relevant countries and in the world will increase to 14 BtCO_2eq_ and 19 BtCO_2eq_ by 2035, with an annual growth of 2.39% and 1.71%, respectively. In summary, the current study’s findings affirm the usefulness of trend analysis in deriving models to estimate greenhouse gas emissions associated with coal combustion.

## Introduction

**Fig. 1:**
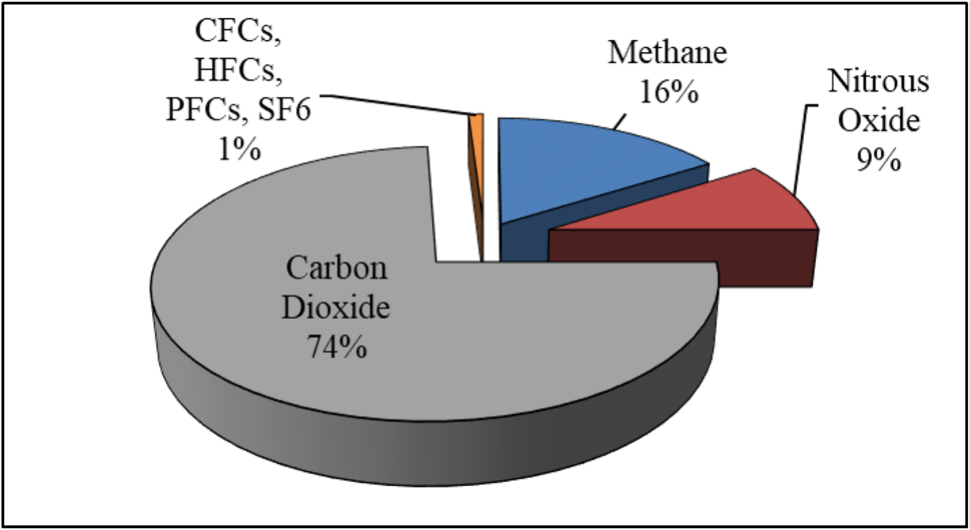
Contribution of the gasses to GHG emissions (Karakurt et al. 2011)

Economic growths and rises in anthropogenic activities contribute to the release of a complex mix of GHGs, trapping the heat and modifying the Earth’s climate (Adam and Apaydin 2016). The principal gasses, responsible for these modifications, include CO_2_, CH_4_, N_2_O, SF6, HFCs, PFCs, and CFCs (Keerthana et al. 2023). Among the GHGs, CO_2_ is primarily to blame for global climate change and global warming (Fig. 1).

As one of the primary causes of GHG emissions globally, FFs currently provide around 82% of the energy supply worldwide, with oil comprising almost 32%, followed by coal (27%) and natural gas (24%) (EI 2024). This dependence on FFs is what drives the majority of the world’s GHG emissions, especially CO_2_ emissions (Li et al. 2024). Various international agreements and organizations, the best known as Kyoto Protocol and Paris Climate Agreement, have been made for reducing and/or replacement of the FFs and therefore GHG emissions so far (Karountzos et al. 2023). However, countries continue to use the FFs due to factors such as the unsteady processes experienced, the instability in the prices of energy resources, the inability to supply energy resources sufficiently and punctually, and other economic concerns. As a result, the average GHG, hence CO_2_ emissions have increased over time in the atmosphere. In 2022, the world’s total GHG emissions reached 54 BtCO_2eq_, of which 38.5 BtCO_2eq_ or roughly 72% was CO_2_ emissions. The five largest emitters, which were China (29.16%), the USA (11.18%), India (7.33%), European Union (6.67%), and the Russian Federation (4.79%), were responsible for 59.14% of the worldwide GHG emissions. In case of total CO_2_ emissions, the USA ranked second at 12.59%, behind China with a 32.88% emission rate. China and the USA were followed by European Union, India, and the Russian Federation at rates of 7.27%, 6.99%, and 4.96%, respectively. These five largest CO_2_ emitters contributed a total of almost 65% global CO_2_ emissions (EDGAR 2024). On the other hand, in 2022, GHGs from energy sector amounted to 36.39 BtCO_2eq_, 33.8 of which resulted from fuel combustion. In other words, fuel combustion was responsible for greater than 95% of the energy-related GHG emissions. Based on the fuel type, FFs constituted the vast majority of them. That is, global energy-related GHG emissions were dominated by coal (over 43%), followed by oil and natural gas, with almost 31% and 22%, respectively (Ritchie et al. 2020; IEA 2022).

As can be appreciated, global warming and climate change are one of the main environmental issues of today. This issue becomes more important especially for developing and emerging countries such as Brazil, Russian Federation, India, China, South Africa, and Turkiye (known as BRICS-T), since the economic development and growth of such countries brings with it an increase in the GHG emissions, especially CO_2_. Thus, researchers and policy makers around the world are increasingly emphasizing the importance of prioritizing the reduction of the GHG emissions on a national, regional, and global levels (Gu et al. 2018; Yang et al. 2021). In addition, it is true that estimating future GHG emissions as precisely as feasible is of great importance from a theoretical and practical perspective for all the national and/or regional policymakers in the disciplines of economics, science, and environment. Such approach will help to establish the suitable measurement techniques that can be taken against the mitigation of the GHG emissions. In this framework, the current research aims at deriving models in order to estimate the GHGE-CC in the BRICS-T countries. TA is employed for deriving the linear and non-linear predictive models, depending on historical trend. The TA is less complex than other techniques, requiring significantly more parameters. It puts out the notion that readers may predict what will happen in the future by looking at what has already happened in the past. Additionally, the greatest benefit of this technique is its simplicity since predictions can be made using any available data (Kone and Buke 2010; Celiker et al. 2021). Other superiorities of the TA can be listed as:

- Almost all statistical software offers a regression toolbox, including the TA tab. This regression toolbox makes it feasible to use the TA to derive linear and non-linear predictive models.
- Because the dependent variable is expressed as a function of just one independent variable, the derived model is more straightforward and understandable.
- All AI-based approaches include “a black box” which implies that the structure of the function needs to be approximated when building a predictive model. However, the TA does not have such a feature.
- Applying the equation to a scenario or prediction is rather simple once the predictive models are derived.

The current research, the first of which consists of an introduction, is structured around seven sections. “Geographic and socioeconomic indicators and energy profile of the BRICS-T nations” section gives the key indicators of the BRICS-T nations. “An overview of the peer-reviewed literature” section reviews shortly the relevant literature and identifies the research gaps. “Modeling approach” section delineates the modeling approach. “Results and discussion” section indicates the results and discussion. “Conclusions” section summarizes the research with the concluding remarks. Limitations and future work are finally given in the “Limitations of the current study and future work” section.

## Geographic and socioeconomic indicators and energy profile of the BRICS-T nations

**Fig. 2:**
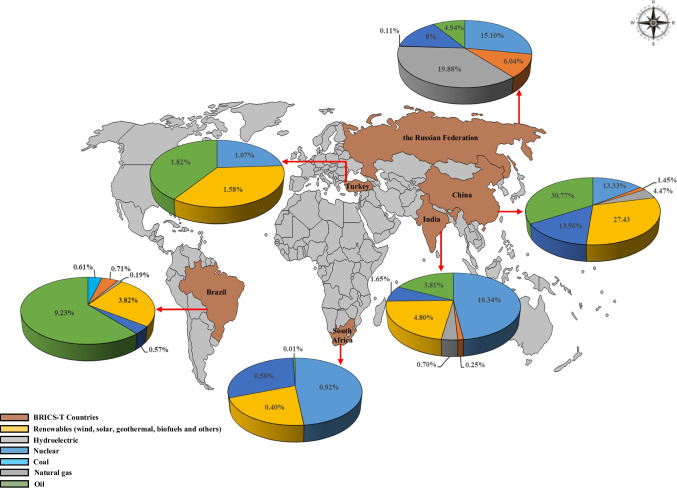
Geographical locations and energy reserves of the BRICS-T nations (EI 2024)

Propounded in 2001 by Jim O’Neill, BRICS stands for a group of five developing nations, such as Brazil, Russia, India, China, and South Africa. Initially, the association was a four-state international organization, as BRIC in 2006, then South Africa became a member in 2010 following the first two summits of the founding four nations (Ibrahim and Ajide 2021). As of 2024, a total of 5 more countries—Egypt, Ethiopia, Iran, United Arab Emirates, and Saudi Arabia—have officially participated in this group of countries, which have been defined as BRICS since 2010: Although the new name of the group is unknown, it is estimated that it will be “BRICS + ” with the new members (Ergezer 2024). In spite of the non-member of the group officially, Turkiye which is an active player in the region exhibits a pattern that is comparable to that of the BRICS nations. Hereafter, BRICS-T countries will be used as an acronym in the current research. BRICS-T nations are extremely influential on today’s world given that they include more than 41% of all people worldwide and govern nearly 27% of the global economy with their over 3 billion people and 27 trillion US dollars GDPs, respectively. Additionally, geographically (see Fig. 2), the BRICS-T nations occupy a total land space of almost 40 million square kilometers or over 30% of global land surface. As of July 2023, India had the largest growth rate with 7.01% annual growth rate, while the lowest rate belonged to South Africa among the BRICS-T economies. In addition, almost all BRICS-T countries are projected to advance their current GDP positions in a high place by 2050 (Table 1) (Siddiqui 2016; Tian et al. 2020; Anser et al. 2021).

**Table 1:** Key indicators of the BRICS-T countries (PwC 2022; WBI 2024)

Countries	TP^1^ (million)	GDP^2^ (billion $)	Land area (sq. km)	Growth rate^2^ (%)	HDI^3^	Projected GDP rankings in the world	
						2030	2050
Brazil	216.422	1920	8,358,140	2.90	0.76	6	5
The Russian Federation	144.444	2240	16,376,870	− 2.07	0.82	7	6
India	1428.627	3385	2,973,190	7.01	0.64	3	3
China	1425.671	17,963	9,388,210	2.99	0.76	1	1
South Africa	60.414	405	1,213,090	2.04	0.71	30	27
Turkiye	85.816	905	769,630	5.57	0.83	14	14
BRICS-T total	3361.394	26.818	39,079,130	–	–	–	–
The world	8113.670	101.003	127,343,220	3.08	–	–	–

^1^As of June 2024

^2^As of July 2023

^3^Human Development Index

HDI is ≥ 0.8 ⇒ very high. HDI is 0.70–0.79 ⇒ high; HDI is 0.55–0.69 ⇒ medium; HDI is ≤ 0.54 ⇒ low

**Fig. 3:**
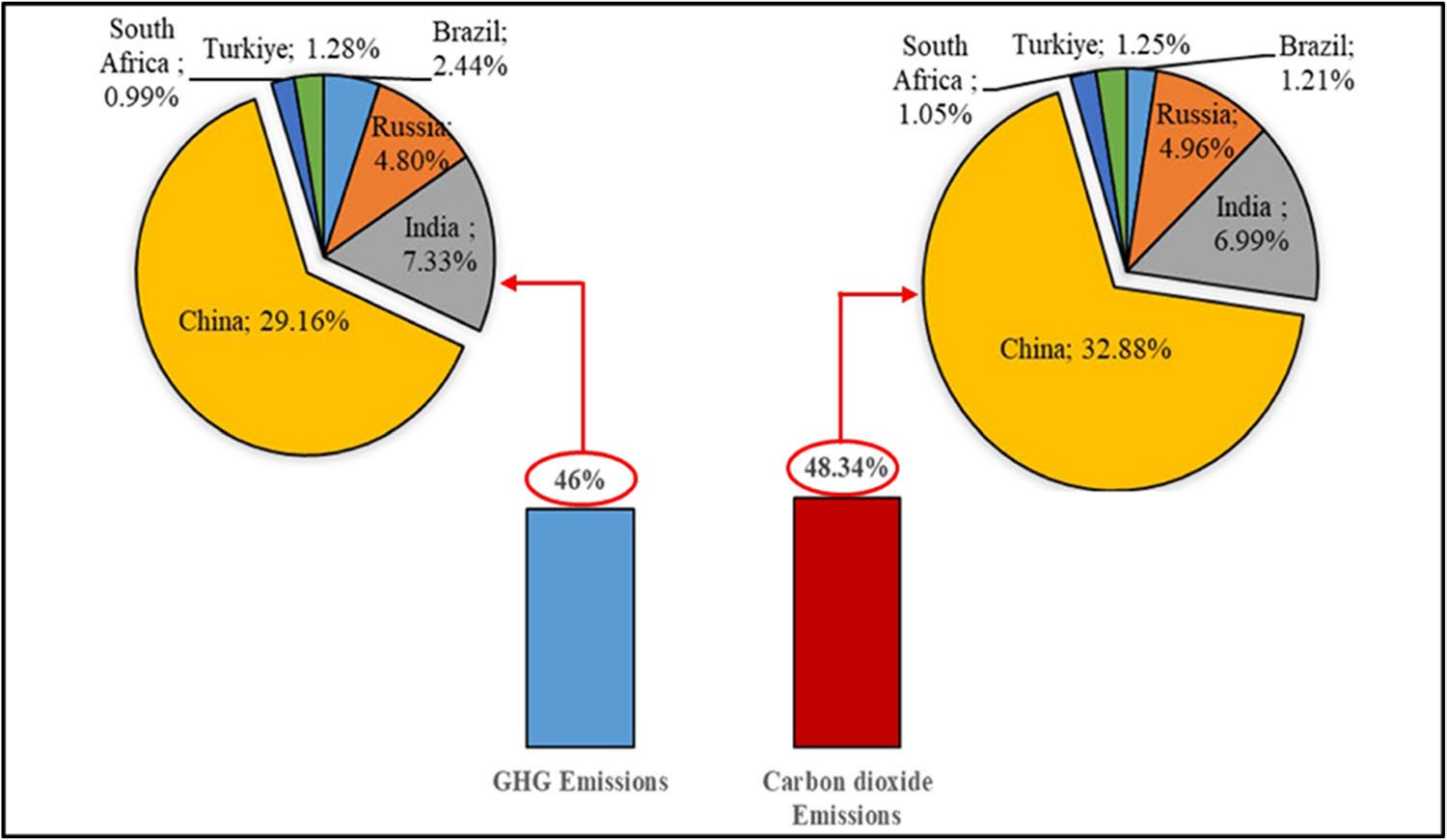
Shares of the GHG and CO_2_ emissions of the BRICS-T nations in the world (Ritchie et al. 2020; EDGAR 2024)

As the developing and emerging economies, BRICS-T countries have also substantial place in terms of their energy profiles in the world. According to EI (2024), in 2023, these countries held around 8.5%, 25%, and 40% of the proven oil, natural gas, and coal reserves worldwide, respectively. In addition to the FFs, they had also significant energy sources such as nuclear, hydroelectric, and renewable (Fig. 2). Aside their significant energy resources worldwide, the BRICS-T’s economic growth and the increased public awareness of the group draw attention to the ongoing increase in their PEC. For example, in 2023, total PEC of the BRICS-T reached to almost 277 exajoule while the world PEC was roughly 620 exajoule, a whopping 43% of all PEC worldwide. China, India, and the Russian Federation among the BRICS nations, they stood out as top three principal nations influencing these rates. On the other hand, a huge majority of the PEC in the BRICS-T nations was met by FFs (almost 82%) in 2023. Among these fuels, coal accounted for roughly half (46%) of the PEC in the BRICS nations, while the hydroelectric, renewable, and nuclear power sources came next respectively in PEC of the group (EI 2024). The BRICS-T nations have currently seen the recent rapid economic expansion, and they have contributed greatly to the growth of the world economy. However, serious environmental problems have also resulted from this rapid economic development like increases in the amount of GHG, thus CO_2_ emissions. For instance, in 2022, total GHG emissions of the group amounted to 24.7 BtCO_2eq_, 18.6 of which was CO_2_ emissions. In other words, almost 46% and 48% of the of the world GHG and CO_2_ emissions originated from BRICS-T countries, respectively (Fig. 3). It can be followed from Fig. 3 that China and India are two largest contributors to both GHGs and CO_2_ emissions whereas South Africa is the least one among the countries in the group.

## An overview of the peer-reviewed literature

Since it accounts for the majority of GHG emissions, in comparison to other GHGs, CO_2_ emissions have received a great deal of attention either by academicians, policy makers, or other stakeholders from all over the world. Therefore, to monitor and estimate GHG and thus CO_2_ emissions, many scholars have proposed various innovative models using from the statistical methods to soft computing techniques at nationwide, regionally, and internationally. As a rising economic power, there is also an extensive, reliable, and growing literature on the BRICS-T countries either as a group or at the individual country level with these methods/techniques. Table 2 provides an overview of current studies on the estimating of GHG emissions in the relevant literature. This overview focuses only on studies conducted for BRICS-T countries on a group and/or individual basis. Typically, Table 2 clearly illustrates that time series, AI-based algorithms, and various mathematical models are employed to estimate the GHG emissions of the BRICS-T countries. Crucially, majority of the researches in the existing literature, some of which are given in Table 2, have concentrated on the total amount of GHG emissions, or just the buildup of CO_2_ emissions in the relevant nations. In addition, the relevant studies have yielded different results due to variations in time periods, models, methodologies, socioeconomic variables, and geographic locations. Therefore, predictive models which are developed with multivariate parameters are essential for future environmental concerns in the making of laws governing regional, global, and national policies. That is, new, straightforward, and understandable predictive models in the relevant sector are still needed. In this way, it can be possible to understand the environmental concerns’ dynamics, climate change’s physical and chemical components more effectively. Additionally, this approach can provide tools to account and achieve for anthropogenic GHG sources increasing the greenhouse effect and awareness of the true impact that human activities have on natural environments, respectively. On the other hand, while the majority of artificial intelligence techniques have the benefits of precisely representing long-term trends phenomenon, they have certain drawbacks in the development of the models and applied in real-world scenarios. In this case, simpler and less precise modeling methodologies may be more suitable if the prediction module is only a component of a more complex planning tool (Bianco et al. 2009). Considering the necessity of predictive models and the lack of prior research on the modeling and estimating the GHG emissions for the BRICS-T nations, this work attempts to fill this gap in the literature. In fact, the aim of the current research is twofold: (i) to derive simple, easy to understand, and practically usable predictive models and (ii) to estimate the future GHGE-CC. The present study adopts BRICS-T nations as the case for the following reasons: (i) in terms of population, economic development, and growth, they are thought to be the countries growing at the fastest rates in the world; (ii) due to the fact that their manufacturing, mining, and construction sectors use a lot of energy, their carbon emissions have increased as their economies have grown and thus, they face serious environmental problems (for example, they include the world’s largest emitters of GHGs and CO_2_) (Cowan et al. 2014); and (iii) as the most carbon-intensive fuel, they include the world’s first and second coal producers, while the first, second, and fifth coal consumers (namely China, India, and the Russian Federation) (Table 3). Also importantly, the ability of TA to model and predict GHGE-CC has not, to the best of the authors’ knowledge, been the subject of any currently published study. Thus, this paper employs, for the first time, TA for developing statistical models to estimate the GHGE-CC. In conclusion, this study stands out as it focuses on the modeling and estimating the GHGE-CC of the BRICS-T countries using the TA.

**Table 2:** An overview of current studies on the estimating of GHG emissions

Authors	Method/technique	Cases	Variables	Target	Period
Pao and Tsai (2010)	PC	BRIC	CO_2_ emissions, EC, GDP	Dynamic causal relationships	1971–2005 1990–2005 (for the Russian Federation)
Kone and Buke (2010)	TA	Selected eleven countries	CO_2_ emissions, EC	Deriving predictive models	1971–2007
Pao and Tsai (2011a)	PC	BRIC	CO_2_ emissions, EC, FDI, GDP	Estimating dynamic relationship	1980–2007 1992–2007 (for the Russian Federation)
Pao and Tsai (2011b)	GPM	Brazil	CO_2_ emissions, EC, GDP	Examining dynamic relationship	1980–2007
Li and Lin (2015)	DDF	China (30 provinces)	CO_2_ emissions, EE	Measuring the energy efficiency performance	1997–2011
Hamzacebi and Karakurt (2015)	GPM	Turkiye	Energy-related CO_2_ emissions	Estimating CO_2_ emissions	1965–2012
Aydin (2015a)	RA	Turkiye	TP, GDP, ANEC, CRWC, FFC	Developing predictive models	1971–2010
Pabuccu and Bayramoglu (2016)	ANN	Turkiye and EU-28	GDP, EP-EC, EU-T, GHG emissions	Forecast the CO_2_ emissions	1990–2015
Ozceylan (2016)	PSO, ABC	Turkiye	EC, GDP, TP, NMV	Forecast the CO_2_ emissions	1980–2008
Dong et al. (2017)	AMG	BRICS	Per capita CO_2_ emissions, GDP, NGC, REC	Scrutinizing the relationship	1985–2016
Ayvaz et al. (2017)	DGMs	Turkiye, total Europe, and Eurasia	CO_2_ emissions	Estimating energy-related CO_2_ emissions	1965–2014
Haseeb et al. (2018)	EKC	BRICS	Financial development, globalization, CO_2_ emissions	Examining the effects of the parameters on CO_2_ emissions	1995–2014
Zhu et al. (2018)	PQR	BRICS	Urbanization and income inequality	Empirically examining the effects the parameters on CO_2_ emissions	1994–2013
Ummalla et al. (2019)	PQR, ARDL	BRICS	HPEC, GDP, CO_2_ emissions	Investigating the effects of HPEC on GDP and CO_2_ emissions	1990–2016
Zhang and Wang (2019)	CCEMG	BRICS	Growth of the service sector, REC, CO_2_ emissions	Investigating the effects of parameters on CO_2_ emissions	1996–2017
Sahin (2019)	TA	Turkiye	EG and CO_2_ emissions	Estimating the EG	2006–2016
Akram et al. (2020)	NDHPC, NPADRL	BRICS	EE, RE, CO_2_ emissions	Investigating the asymmetric impacts of EE, RE, and other factors on CO_2_ emissions	1990–2014
Liu et al. (2020)	3SLS	Brazil, India, China, South Africa	Real output, REC, and CO_2_ emissions	Investigating the relationships between parameters and CO_2_ emissions	1999–2014
Raghutla and Chittedi (2020)	MCOLS, HPCT	BRICS	Financial development, urbanization, EC, and CO_2_ emissions	Investigating the short- and long-term links between parameters and CO_2_ emissions	1998–2016
Tian et al. (2020)	MRIO	BRICS	CO_2_, SO_2_, water, land, energy, and material footprints	Revealing the role of BRICS countries at global economic and environmental resource	1995–2015
Uzlu (2021)	ANN-GWO, ANN-ABC, and ANN-TLBO	Turkiye	GDP, EC, population, UR, REP, GHG emissions	Forecasting the GHG emissions	1990–2017
Ganda (2021)	PTM	BRICS	FDI, domestic credit to the private sector, energy supply and human capital, CO_2_ emissions	Investigating the relationship between the parameters and CO_2_ emissions	2000–2018
Zhao et al. (2021)	NARDL	BRICS	Geopolitical risk, EC, and CO_2_ emissions	Examining the asymmetric influence of geopolitical risk on EC and CO_2_ emissions	1985–2019
Xu et al. (2021)	BR-AGM (1,1) GM(1,1) OGM(1,1) SVR	China	Coal based EC, GHG emissions	Forecasting the GHG emissions	2000–2016
Sun and Ren (2021)	EEMD-PSOBP and 14 comparative models	China	Daily CO_2_ data	Forecasting of short-term CO_2_ emissions	2019–2020
Bakay and Ağbulut (2021)	ANN, DL, SVM	Turkiye	EP, GHG emissions	Forecasting of GHG emissions	1990–2018
Bakır et al. (2022)	MPA, LSA, EO SOS, BSA	India	REG, electricity generation from coal, gas, and oil, GDP, TP	Forecasting of GHG emissions	1990–2018
Ağbulut (2022)	DL, SVM, ANN	Turkiye	GDP, TP, vehicle—km, year	Forecasting the transportation-based CO_2_ emissions and energy demand	1970–2016
Kumar et al. (2022)	IDW, kriging, spline	India	SO_2_, NO_2_	Predicting the concentration of air pollutants and assessing the interpolation techniques	A monthly spatial average for 2012
Abbas et al. (2022)	SGPRT, LNCT	BRICS	REP, market regulation, environmental innovation, CO_2_ emissions	Examining the impact of parameters on CO_2_ emissions	1990–2020
Kartal (2022)	MARS	China, India, Japan, the Russian Federation, and the USA	PEC, OC, NGC, NUC, REC, CO_2_ emissions	Examining the impact of parameters on CO_2_ emissions	1965–2019
Shi et al. (2022)	Tapio’s decoupling elasticity model	16 districts of Beijing (China)	EC, CO_2_ emissions	Analyzing the decoupling status of CO_2_ emissions	2006–2017
Oladunni et al. (2022)	STIRPAT	South Africa	TP, GDP, energy intensity, urbanization, INFI, FC, FT, PV	Evaluating the GHG emissions and their influences in the transport sector	2011–2020
Iqbal et al. (2023)	ARDL, PMG, MG, the Dumitrescu-Hurlin panel causality tests	BRICS	CO_2_ emissions, REC, FDI, exports, GDP	Determining the effects of parameters on GDP	2000–2018
Ahmed et al. (2023)	SVM, ANN, LSTM	China, India, the USA, and Russia	EC, GHG	Investigating the EC and its trend in GHG emissions	1980–2018 1992–2018 (for the Russian Federation)
Karakurt and Aydin (2023)	RA	BRICS and MINT	TP, UP, GDP, and FF-CO_2_ emissions	Developing predictive models to forecast the FF-CO_2_ emissions	1980–2015

**Table 3:** World top five energy consumers, coal producers, and coal consumers in 2023 (EI 2024)

Energy consumption			Coal production			Coal consumption		
Country	Exajoule	Share (%)	Country	Exajoule	Share (%)	Country	Exajoule	Share (%)
China	170.74	27.56	China	93.10	51.94	China	91.94	56.05
USA	94.28	15.22	India	16.75	9.34	India	21.98	13.40
India	39.02	6.30	Indonesia	15.73	8.78	USA	8.20	5.00
The Russian Federation	31.29	5.05	USA	11.84	6.60	Japan	4.54	2.77
Japan	17.40	2.81	Australia	11.66	6.51	Indonesia	4.32	2.63
The world	619.63		The world	179.24		The world	164.03	

## Modeling approach

### Variable and data description

**Fig. 4:**
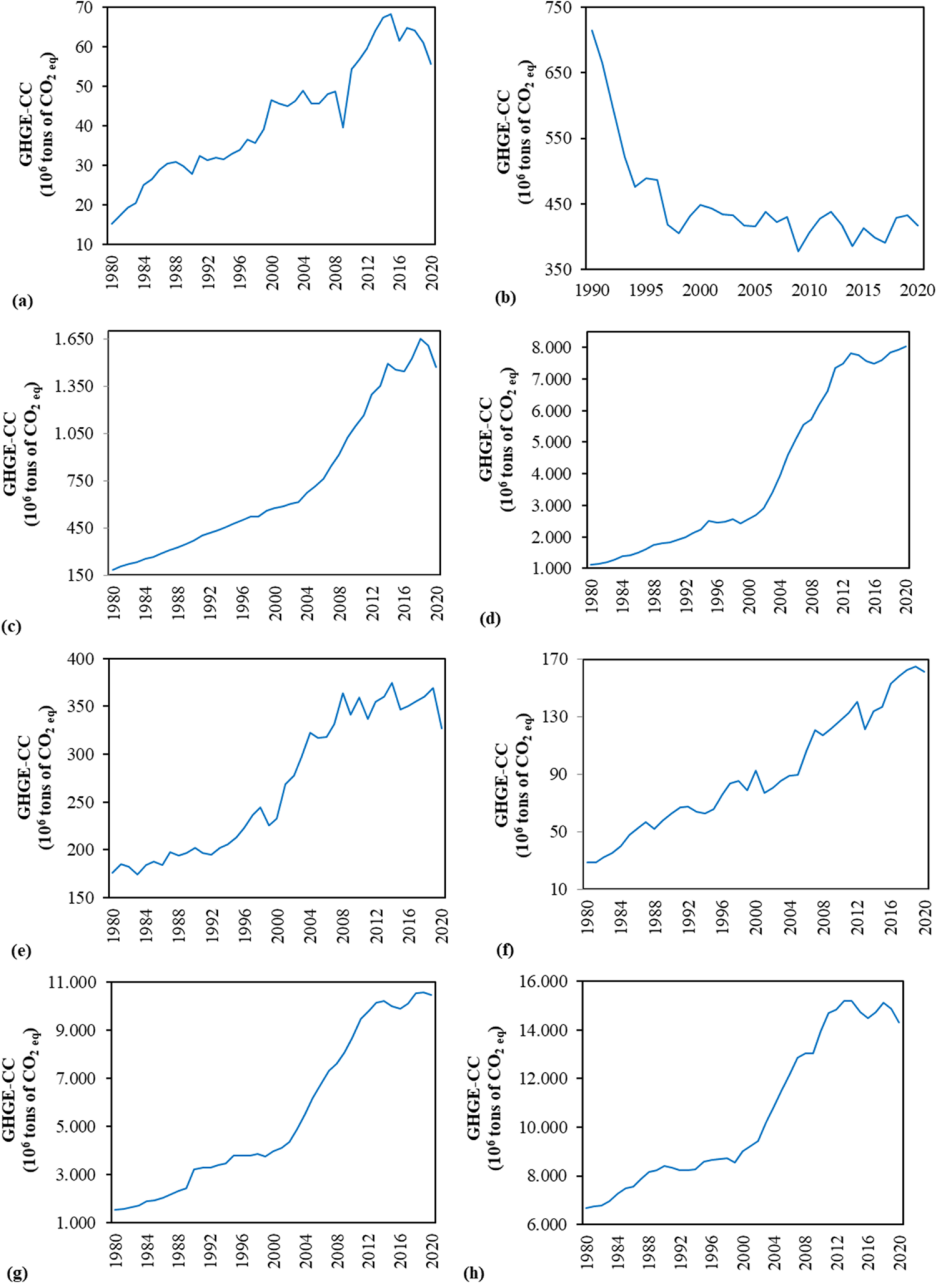
Historical trend of the GHGE-CC in the BRICS-T nations and world (sourced from IEA 2022 GHG emissions from energy—coal combustion) [**a** Brazil, **b** the Russian Federation, **c** India, **d** China, **e** South Africa, **f** Turkiye, **g** the BRICS-T total, and **h** the world]

**Fig. 5:**
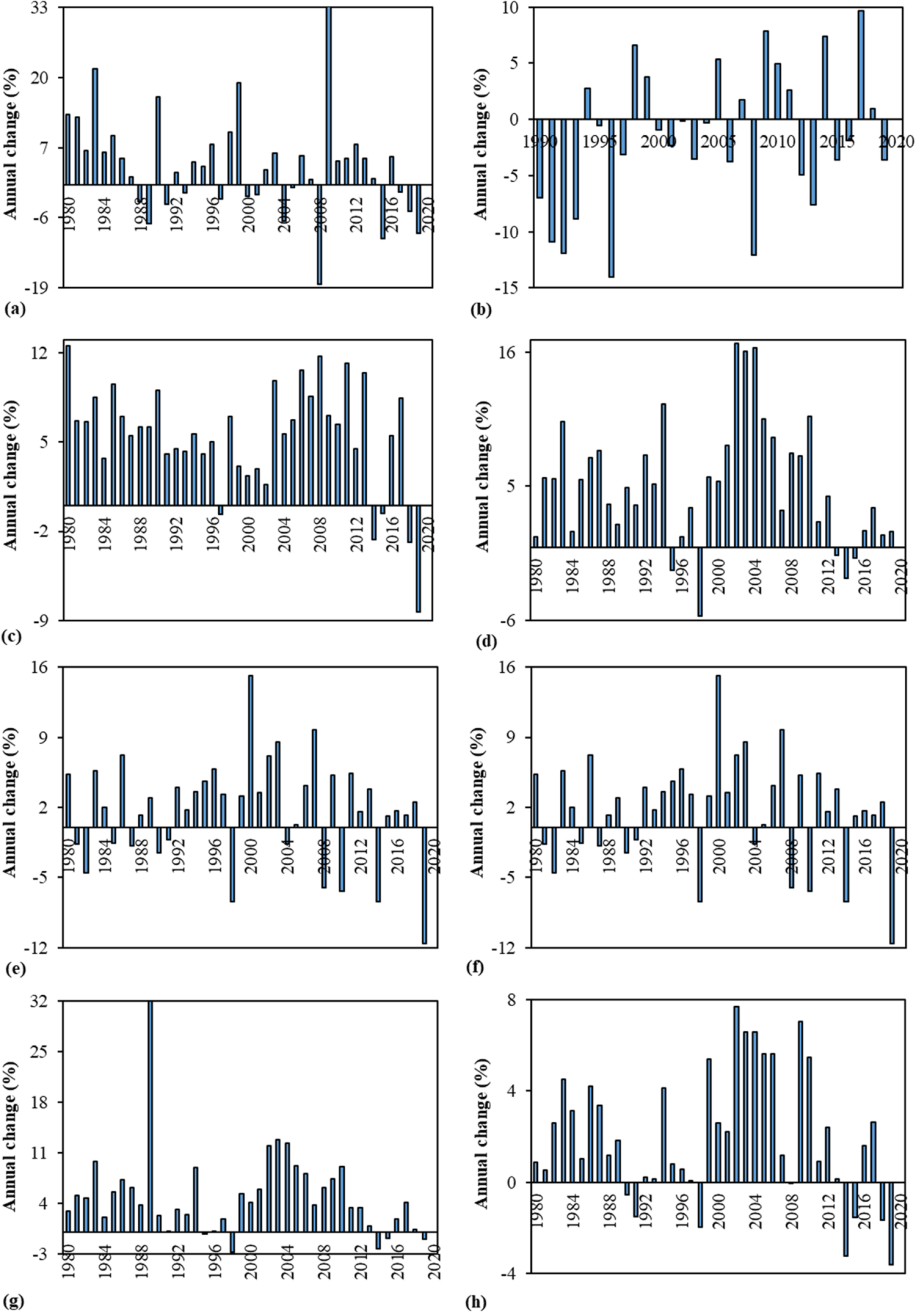
Annual changes in the GHGE-CC of the BRICS-T countries and world during the studied period [**a** Brazil, **b** the Russian Federation, **c** India, **d** China, **e** South Africa, **f** Turkiye, **g** the BRICS-T total, and **h** the world]

Since they account for a large percentage of GHG emissions associated with FF combustion, the GHGE-CCs, which are dominated by CO_2_, with CH_4_, NO_x_, and SO_2_, making lesser contributions to GWP, weighted GHG emissions as well as the particles of PM_2.5_–PM_10_, O_3_, VOCs, and over eighty hazardous air pollutants (e.g., lead, arsenic, and benzene) (Whitaker et al. 2012; Filonchyk and Peterson 2023), were chosen as the dependent variable in the current study. These gasses are mostly emitted when coal is burned in coal power plants to produce electricity. Annual data of the related emissions have been officially and freely sourced from International Energy Agency (IEA 2022) for the period of 1980–2020, for both the BRICS-T nations and entire world. It is essential to note that the data for the Russian Federation are accessible starting in 1990 because the country was founded in the 1990s as a result of the collapsing of the Soviet Union. That is, the Russian Federation’s beginning year was set to 1990. Historical trends of the GHGE-CCs in the BRICS-T nations and world are illustrated in Fig. 4a–h. Except the Russian Federation, it illustrates that the GHGE-CCs of the related economies and world have shown an upward trend, with the average annual changes of 3.72% for Brazil, 4.83% for India, 5.19% for China, 1.76% for South Africa, 3.53% for Turkiye, 4.10% for BRICS-T total, and 1.84% for the world during studied period. In this period, among the group members, only the GHGE-CC of the Russian Federation showed a decreasing trend annually 1.58% (Fig. 5a–h). It is evident from Fig. 5 that the largest annual average growth belonged to China and India at around 5%, while the lowest ones were South Africa and the world at 1.76% and 1.84%, respectively. Taken into account overall, it can be inferred from Fig. 5 that almost all countries in the group had the annual average growth rates, which were higher than the world’s experienced during the selected period, except the Russian Federation, which is the only country that experienced annually decline in its GHGE-CC. Despite the upward trend in the GHGE-CCs, in 2009, all countries in the group and the world experienced a decrease in the related emissions as a result of the global economic crisis. And as a result of the economies’ post-crisis rebound, the emissions rose once more in 2010–2020. Nonetheless, the pace of increase in the emissions was substantially slower than the rate of rise in the emissions in the 1990s. On the other hand, it is worth to note that the GHGE-CC had declined since the early 1990s for the Russian Federation, when the nation’s economic and social structure shifted from the golden periods of the former Soviet Union to the recently formed state. In other words, emissions had significantly decreased in the 1990s, which had been linked to the nation’s overall economic status. Between 1999 and 2008, when the economy was generally recovering, emissions showed fluctuated growth, although it was significantly slower than the rate of decline in emissions in the 1990s. It was most likely caused by the worsening economic conditions that persisted by the early 2000s, not by increased environmental concerns and demands for a better environment.

### Descriptive statistics

The figures in Table 4 display the chosen variable’s descriptive statistics. The data in Table 4 show that the mean of almost each country is comparatively higher than the standard deviations. For example, the GHGE-CC of China is 3329.313 while its SD is 2156.653. Similarly, the GHGE-CC of the Russian Federation is 461.196, whereas its SD is 82.309. The difference is significantly large and displays the most associated variation as measured by the CV. As a result, it is seen that the means are relatively higher than the SDs for all countries in the group. This is a good indication for the data set being homogenous and consistent. Additionally, for an observed data set to be symmetrical or regularly distributed, the general idea is that the relevant values should be zero, based the kurtosis and skewness values. But some scientists claim that if both readings were within ± 1.5, the observed series can also be thought of as having a normal distribution (Urbano 2013; Erbay and Beydogan 2017; George and Mallery 2021). Specifically, the data series of all countries in the group including the BRICS-T total and world indicate positive skewness demonstrating a thicker tail on the right side. Furthermore, it is seen that all data series in the group again including the BRICS-T total and world validate that the kurtosis curve is platykurtic because they are lower than the usual value. Thus, it can be concluded that the skewness and kurtosis values in Table 4 approve normal distribution of the series. The results of the skewness and kurtosis are consistent with the JB test for normality, which offers convincing evidence that all observed series exhibit a normal distribution due to the largest *p* values (*p* > 0.05) of the JB test. As a result, it may be said that the data largely supports one of the core presumptions for the regression model, as proposed by Ostrom (1978).

**Table 4:** Descriptive statistics for the GHGE-CC in the BRICS-T countries

Country	Variable	Mean	Min	Max	SD	CV (%)	Skewness	Kurtosis	JB test
Brazil		38.264	15.110	67.430	13.102	34.241	0.352	− 0.385	0.625
The Russian Federation		461.196	377.420	714.790	82.309	17.847	2.056	1.018	0.010
India		600.649	183.250	1494.920	350.405	58.338	1.057	0.339	0.035
China	The GHGE-CC	3329.313	1128.660	7804.880	2156.653	64.778	0.985	− 0.428	0.052
South Africa		253.285	173.920	374.370	68.802	27.164	0.512	− 1.391	0.114
Turkiye		79.419	28.30	140.120	32.255	40.614	0.294	− 0.851	0.458
BRICS-T total		4630.353	1531.250	10,211.440	2708.880	58.503	0.845	− 0.498	0.104
The world		9814.579	6681.050	15,204.940	2689.562	27.404	0.871	− 0.597	0.084

### Methodology

**Fig. 6:**
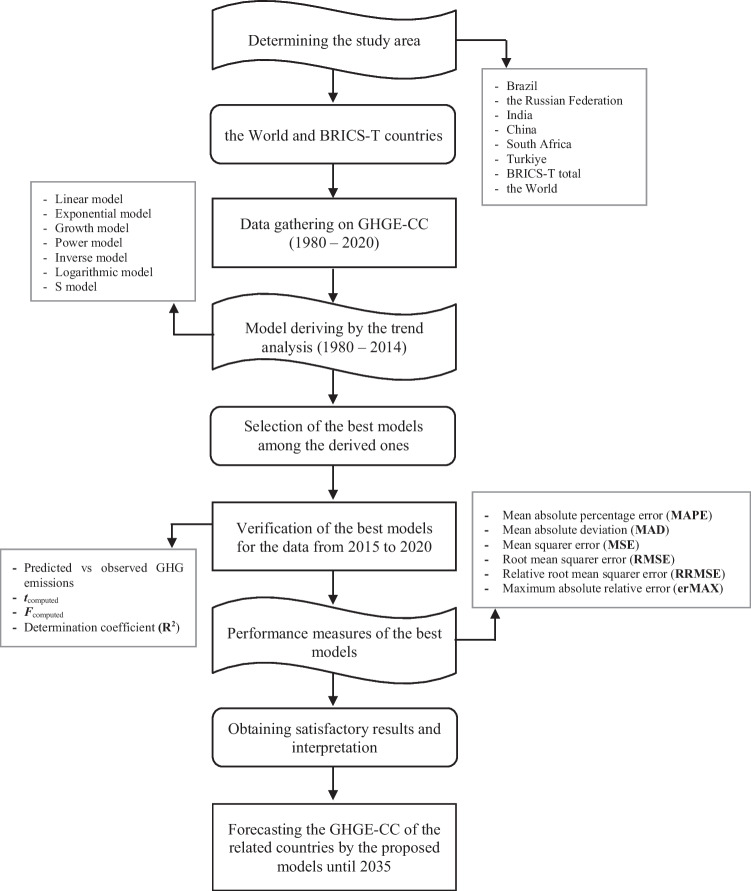
Applied methodology

In the current research, the TA was utilized to model the GHGE-CCs. The TA uses the historical trend of a dependent variable for deriving models to estimate future values of the chosen variable. Hence, it is anticipated in the analysis that the GHGE-CC would continue to follow the historical trend that has previously been observed. As it has been reported in the previous sections as well, this method’s key benefit is its simplicity and estimates can be made using any accessible data (Aydin et al. 2015; Aydin 2015b; Kok and Benli 2017). In the current study, predictive models were derived based on the linear, logarithmic, power, exponential, inverse, growth, and S regressions whose mathematical expressions are given in Table 5. The GHGE-CCs’ annual data were divided into two groups: the data used to train the model from 1980 to 2014, which accounted for 85% of the total data, and the data used to test the model from 2015 to 2020 (14% of the total data). SPSS statistical software, which provides a choice of regression toolbox, was utilized for deriving the models. Once the model is derived, the goodness of the proposed model was statistically verified by taking into account the *R*^2^, the *t*-test, the *F*-test, and the predicted versus the observed data. The *R*^2^ is often used in statistics for verification and measurement of the performance of the models. It serves as a benchmark for evaluating the model’s precision and provides the percentage of one variable’s variance that can be predicted from another. Greater model reliability is shown by high *R*^2^ values (Aydin 2015c; Despotovic et al. 2015). The *F*- and *t*-tests play significant roles in statistical inference, directly affecting model coefficients, confidence ranges, and, ultimately, the results of hypothesis testing. The computed and tabulated *t* values are compared using the null hypothesis in the *t*-test. The correlation is significant if the computed *t* value is higher than the tabulated value. To evaluate the significance of the suggested models, an analysis of variance was also used, at the 95% confidence level. When performing the variance analysis, if the computed *F* value is larger than the tabulated *F* value, confirming the statistical significance of the proposed models (Uma et al. 2011; Aydin 2015b). Moreover, the estimating performances of the derived models were also measured through various statistical indices such as the MAD, MSE, RMSE, RRMSE, erMAX, and MAPE. Regardless of performance criteria, generally, the lower the value of the criteria, the better the fitted curve matches the real data (Singh et al. 2009; Paiva et al. 2021). In other words, the proposed model is more precise when the indicators are smaller. On the other hand, representing the errors associated with the models, the MAD and MSE are two measures for the absolute projected error’s average size (Bianco et al. 2014), while the RMSE measures the variance of estimated values around the measured data and offers information on the performance over the short term. Determined by dividing the RMSE to the average value of the measured data, the RRMSE is an indicator of the overall relative accuracy of a model. The performance criteria, used for measuring the estimating accuracies of the derived models, can be computed as given in Table 6. Furthermore, the present research’s flowchart is displayed in Fig. 6.

**Table 5:** Mathematical expressions of the linear and non-linear models

Model type	Equation
Linear	$y=a+b\cdot (t)$
Logarithmic	$y=(a)+\text{ln}\left(t\right)$
Power	$y=(a{)\cdot (t)}^{b}$
Exponential	$y=(a{)\cdot e}^{b(t)}$
Inverse	$y=(a)+(b)\cdot \left(\frac{1}{t}\right)$
Growth	$y={e}^{[\left(a\right)+\left(b\right)\cdot \left(t\right)]}$
S	$y={e}^{\left[\left(a\right)+\left(b\right)\cdot \left(\frac{1}{t}\right)\right]}$

Where *y* is the GHGE-CC (MtCO_2eq_), *a* is the intercept or the constant, *b* is the slope, and *t* is the year

**Table 6:** Metrics for assessing the models’ efficacy

Mean absolute deviation	$MAD=\left(\frac{1}{n}\sum_{i=}^{n}\left|{X}_{i}-{Y}_{i}\right|\right)$
Mean square error	$MSE=\frac{1}{n}{\sum }_{i=1}^{n}{\left({X}_{i}-{Y}_{i}\right)}^{2}$
Root mean square error	$RMSE=\sqrt{\frac{1}{n}{\sum }_{i=1}^{n}{\left({X}_{i}-{Y}_{i}\right)}^{2}}$
Relative root mean square error	$RRMSE=\frac{RMSE}{\overline{O} }\cdot 100$
Maximum absolute relative error	$erMAX=max\left(\left|\frac{{X}_{i}-{Y}_{i}}{{Y}_{i}}\right|\right)$
Mean absolute percentage error	$MAPE=\frac{1}{n}{\sum }_{i=1}^{n}\left(\frac{\left|{X}_{i}-{Y}_{i}\right|}{{Y}_{i}}\right)\cdot 100$

Where *n* is the total number of data, *X*_*i*_ is the predicted GHGE-CC, *Y*_*i*_ is the actual GHGE-CCs, and $\overline{O }$ is the mean value of observed data

## Results and discussion

### Modeling results and statistical verification

**Fig. 7:**
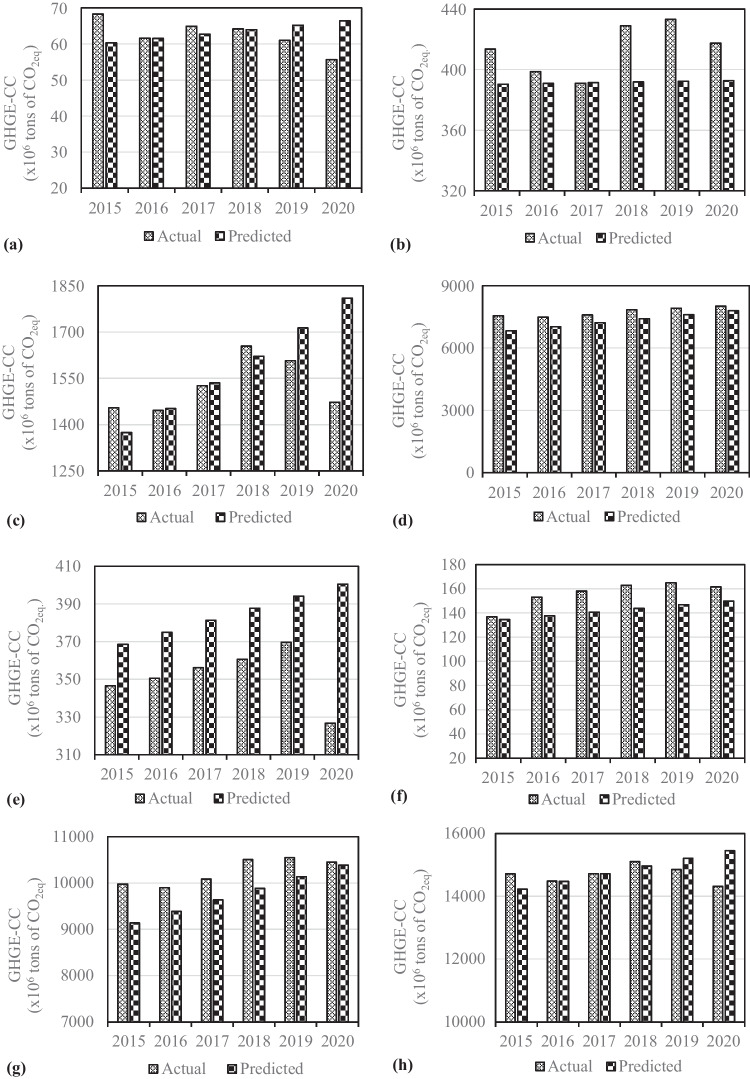
Predicted vs actual values of the GHGE-CC. **a** Brazil; **b** the Russian Federation; **c** India; **d** China; **e** South Africa; **f** Turkiye; **g** BRICS-T total; **h** the world

Based on the statistical results, provided in Table 7, the derived models are listed in Table 8. The models in all equation forms (given in Table 5) were firstly produced for all nations separately in the bloc, BRICS-T total and the world. Then, the models in Table 8 were selected on basis of mainly their *R*^2^ values (models with the highest *R*^2^ values were selected), demonstrating strong associations between the GHGE-CCs and year. It is evident from Table 8 that the derived models for Brazil, China, South Africa, the BRICS-T total, and world are based on the linear function, while those are explained by reverse and growth functions for the Russian Federation and India, respectively. On the other hand, statistical verification results of the derived models are presented in Table 9. It is clearly seen that the derived models for Brazil, South Africa, Turkiye, and the BRICS-T total have *R*^2^ values above 0.90, while those for the Russian Federation, India, China, and the world have *R*^2^ values over 0.85. Both *R*^2^ values indicate a strong relationship between year and the GHGE-CCs, verifying the derived models statistically. In addition, the both *R*^2^ values show that all causes other than the predictors account for at least 0.1% of the variation in the GHGE-CCs for Brazil, South Africa, Turkiye, and the BRICS-T total, whereas 0.15% of the variation in the GHGE-CCs for the Russian Federation, India, China, and the world. However, it is not enough to fit a model based on only *R*^2^ values, providing confidence intervals, or conducting a test to conclude a regression analysis. This provides us only half the story. Thus, we need to look more verification results. Table 9 tells us that the computed *F* and *t* values are higher than the tabulated ones for all models, verifying the correctness of the derived models and the coefficients in the models respectively at the 95% confidence level. Additionally, the predicted vs actual data graphs are illustrated in Fig. 7 as an another statistical verification indicator. As can be seen from Fig. 7, the predicted data by the derived models closely match the actual data, indicating the models’ statistical soundness. As a result, it can be concluded that the derived models appear to be statistically valid based on the study’s conditions.

**Table 7:** Statistics from the derived models

Country	Model	Variable	Coefficients	Standard error	Standard error of estimation
Brazil	Linear	Constant	16.248	1.339	3.876
		Year	1.223	0.064	
Russia	Inverse	Constant	404.737	7.853	31.344
		Year	369.885	30.986	
India	Growth	Constant	5.246	0.024	0.071
		Year	0.055	0.001	
China	Linear	Constant	− 169.719	14.044	839.138
		Year	194.391	289.871	
South Africa	Linear	Constant	138.056	7.276	21.064
		Year	6.402	0.352	
Turkiye	Linear	Constant	24.325	2.642	7.647
		Year	3.061	0.127	
BRICS-T total	Linear	Constant	124.879	305.585	884.630
		Year	250.304	14.806	
The world	Linear	Constant	5401.600	336.791	974.967
		Year	245.166	16.318

**Table 8:** Derived predictive models

Countries	Models
Brazil	$y=\left(16.248\right)+\left(1.223\right)\bullet (t)$
The Russian Federation	$y=\left(404.737\right)+(369.885)/(t)$
India	$y={e}^{[\left(5.246\right)+\left(0.055\right)\bullet (t)]}$
China	$y=\left(-169.719\right)+\left(194.391\right)\bullet (t)$
South Africa	$y=\left(138.056\right)+\left(6.402\right)\bullet (t)$
Turkiye	$y=\left(24.325\right)+\left(3.061\right)\bullet (t)$
BRICS-T total	$y=\left(124.879\right)+\left(250.304\right)\bullet (t)$
The world	$y=\left(5401.600\right)+\left(245.166\right)\bullet (t)$

**Table 9:** Statistical verification of the derived models

Country	Variables	*t*_computed_	*t*_tabulated_	*F*_computed_	*F*_tabulated_	*R*^2^
Brazil	Constant	12.134	1.690	355.425	4.125	0.92
	Year	18.853				
The Russian Federation	Constant	51.539	1.708	142.491	4.240	0.86
	Year	11.937				
India	Constant	215.194	1.690	242.022	4.125	0.88
	Year	46.761				
China	Constant	− 0.585	1.690	191.581	4.125	0.85
	Year	13.841				
South Africa	Constant	18.973	1.690	329.731	4.125	0.91
	Year	18.158				
Turkiye	Constant	9.209	1.690	571.947	4.125	0.95
	Year	23.915				
BRICS-T total	Constant	0.409	1.690	285.812	4.125	0.90
	Year	16.906				
The world	Constant	16.038	1.690	225.739	4.125	0.87
	Year	15.025

### Evaluation of the estimating performances of the derived models

Among the several criterions for performance measures, the MAPE is the best suited to assess the relative error, as input data used for model estimation, pre-processed data and raw data all have different scales. Due to its benefits of scale independence and interpretability, it is classified as one of the most popular metrics of estimating accuracy. Because of its scale-independent and simplicity of interpretation, professionals in the sector also favor the MAPE (Azadeh et al. 2011; Byrne 2012; Kim and Kim 2016). Additionally, while in the same tendencies with other indices, value of RRMSE was also selected providing the decisive index on the derived models’ estimating performance metrics in the present study together with the MAPE values as recommended by other estimating such as Bianco et al. (2010), Li et al. (2013), Bianco et al. (2014), and Kim and Kim (2016). Tables 10 and 11 provide and list the reference table of the MAPE and RRMSE levels and results of the derived models’ performance metrics, respectively. Table 11 makes it clear from the MAPE and RRMSE results that all of the derived models for Brazil, the Russian Federation, China, India, South Africa, Turkiye, the BRICS-T total, and the world may estimate excellent. Besides the MAPE and RRMSE values, other performance indicators are also smaller, indicating the prices level of the derived models for the estimating. Other performance metrics have also low relative errors, as Table 11 demonstrates in addition to RRMSE and MAPE values. This is a reliable indicator of how well the derived models estimate the GHGE-CCs.

**Table 10:** Reference table of the MAPE and RRMSE for the model accuracies (Lewis 1982; Li et al. 2013)

MAPE (%)	RRMSE (%)	Predictive ability
MAPE ≤ 10	RRMSE < 10	Excellent
11 ≤ MAPE ≤ 20	10 < RRMSE < 20	Good
21 ≤ MAPE ≤ 51	20 < RRMSE < 30	Qualified
MAPE > 51	RRMSE ≥ 30	Unqualified

**Table 11:** Performance metrics for the proposed models

Countries	Performance indicators					
	MAPE	MAD	MSE	RMSE	RRMSE	erMAX
Brazil	6.96	4.24	0.56	0.75	1.20	0.19
The Russian Federation	5.25	22.23	1.65	1.28	0.31	0.09
India	6.34	95.27	14.88	3.86	0.25	0.23
China	5.47	420.16	26.47	5.15	0.07	0.10
South Africa	9.52	32.84	4.20	2.05	0.58	0.23
Turkiye	8.80	14.03	1.44	1.20	0.77	0.12
BRICS-T total	4.76	484.03	28.50	5.34	0.05	0.08
The world	2.44	355.61	19.36	4.40	0.03	0.08

Meanwhile, the current study’s methodology or modeling technique does not claim that the TA derives the most accurate models for estimating the output variable. On the contrary, aforementioned, the TA is less complex than other techniques that require significantly more parameters/variables and its greatest benefits are the simplicity, easy to understand and to derive practically usable predictive models. The models’ accuracies are sometimes less than those of the other computing techniques. Despite this, when the results of the current study on the basis of error analysis were compared with the results of some other studies, aiming at modeling and estimating the GHG emissions using different modeling techniques in the relevant literature, it is seen that the suggested models had fewer relative errors. In other words, the proposed models in the current study have less MAPE values indicating that they have high accuracies over other developed models in the existing literature. It can also be inferred from the comparison results that the TA can derive models, having almost the same errors that is obtained with other techniques (Table 12).

**Table 12:** A comparison of the current results with the relevant studies in the published literature

Relevant literature				Current study		
Researcher (s)	Case(s)	Model		MAPE (%)	Case(s)	MAPE (%)
Fang et al. (2018)	China	PSO-GPR		9.13	China	5.47
		GPR		16.89		
		BPNN		29.70		
Adarkwa et al. (2020)	China	DGM		8.30	China	5.47
Qiao et al. (2020)	World	SMOSVM		2.83	World	2.44
Ding et al. (2020)	China	The rolling DGPM (1,N)	M1	1.67	China	5.47
			M2	11.47		
			M3	18.30		
			M4	16.04		
			M5	8.58		
Sun and Ren (2021)	China	EEMD-PSOBP		9.30	China	5.47
Bakır et al. (2022)	India	LSA		5.21	India	6.34
		EO				
		SOS				
		BSA		5.29		
Huang et al. (2022)	China	ENGM(1,4)		0.44	China	5.47
		GM(1,4)		2.03		
		GMC(1,4)		14.45		
		SVR		2.60		
		ANN		3.73		
		ARIMA		24.57		
Ağbulut (2022)	Turkiye	DL		9.81	Turkiye	8.80
		ANN		8.57		

### Estimating results and future projections

**Fig. 8:**
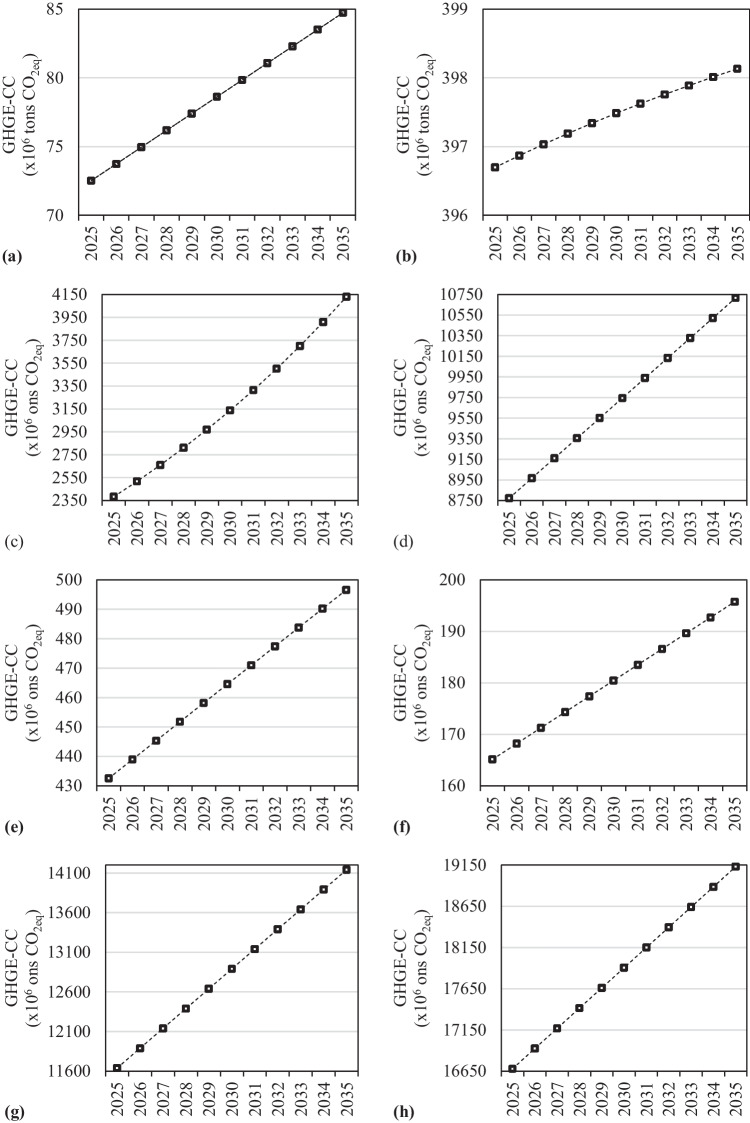
Projected results for the GHGE-CCs

**Fig. 9:**
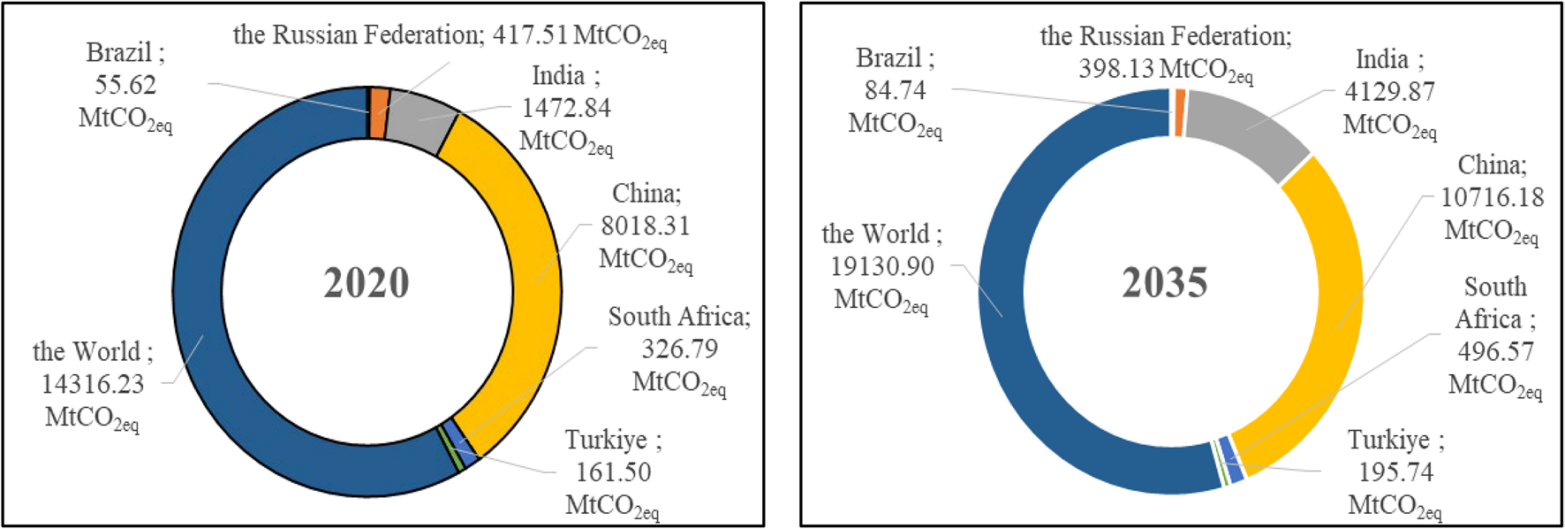
Total GHGE-CCs of the BRICS-T countries and world in 2020 and 2035

Future GHGE-CCs were estimated using the derived models and the results are depicted in Figs. 8 and 9. The related graphs clearly illustrate that the GHGE-CCs of the BRICS-T total will increase from 10.452 MtCO_2eq_ in 2020 to 14.141 MtCO_2eq_ in 2035, with an annual growth of 2.39%. In other words, it is projected that the GHGE-CCs of the BRICS-T total will have increased by around 35% by the year 2035. With the derived model explained by a linear function, almost the same increase (around 34%) in the world’s GHGE-CCs is also expected in 2035 (Fig. 9). In addition, it is evident from the related figures that significant increases are expected for all nations separately in the bloc, aside the Russian Federation, when compared the data 2020 to 2035. For instance, the GHGE-CCs will be considerably high in India. It is anticipated that India’s GHGE-CCs will rise from 1472.84 MtCO_2eq_ in 2020 to 4129.87 MtCO_2eq_ in 2035, representing an annual growth rate of 11.87% and a total change of 180.40% in 2035. With this increase, India will be the country with the largest expected increase of all the countries in the group. India is followed by Brazil, South Africa, China, and Turkiye, with 52.34%, 51.95%, 33.65%, and 21.20% total change rates in 2035, respectively. Despite a modest rising trend between the years in the estimation, a decline in total GHGE-CCs is anticipated in 2035 for the Russian Federation, as compared to the other nations in the group and the global average. In other words, the Russian Federation’s GHGE-CCs are projected to fall from 417.21 MtCO_2eq_ in 2020 to 398.13 MtCO_2eq_ in 2035, indicating a total decrease rate of almost 5%. However, this expected decline rate for the Russian Federation’s GHGE-CCs in 2035 is not as high as the expected increase rates for the GHGE-CCs of other countries in the group.

It is possible to say that the estimated results of the current study are aligned with the future projections of the relevant nations and the world are consistent. For instance, over 70% of India’s power generation is generated from coal, which is the county’s main fuel. Guttikunda and Jawahar (2014) highlighted that the role of the coal in energy balance of India will expand in the upcoming years. In addition, according to Prayas (2011 and 2013), coal will continue to produce a significant amount of power at least through 2030 in India. Given that the significance of pollution coming from coal-fired power plants and health damages throughout the world as well as emitting GHGs and current status of India, where coal is the primary fuel for power generation, it may be concluded that a large percentage of the GHGE-CCs will be expected for the foreseeable future as similar to estimating results of the current study, unless a better option is deployed to manage emissions from these plants. Besides India, a study, measuring key long-term drivers for the energy mix including emissions in another official member country, Brazil, determined that the combustion of fuels for the transportation sector, as well as the combustion of coal, coke, and charcoal, stand out as highly polluting sectors of supply chains in the long term (Lenzen et al. 2013). Thus, one can project that the utilization of coal in these supply chains and adjacent industries will lead to an increase in GHGE-CCs as estimated in the case of the current study. Moreover, as the largest national emitter of GHGs and whose primary energy production and consumption is mainly in coal, China has encouragingly capped the coal consumption in order to mitigate emissions recently (Qi et al. 2018). With slower but better quality economic growth and accelerating the transition to clean energy, it will not be surprising to expect a downward trend in future emission values. Studies modeling and estimating China’s energy-related CO_2_ emissions also state that 2030 will be the peak year (Peters et al. 2015, 2017; Green and Stern 2016; Elzen et al. 2016). Taking current trends into account, it can be stated that China’s emissions would reach a stable plateau with minor fluctuations within the next years. The findings of this investigation, in which it is estimated that the GHGE-CC will increase with a slow growth of 2.42% annually towards 2035, also support these findings for the country. Furtherly, in terms of the Russian Federation’s decreasing trends in the GHGE-CCs in the future, necessitates examining the nation’s primary sources of energy. In 2023, natural gas made around 52% of the energy supplies, followed by oil at roughly 23% and coal at about 12%. Nuclear power dominates the non-fossil fuel energy source (6%), followed by the renewables (6%), majority of which is hydropower (CTR 2023). Here, it is significant to note that around 37% of Russia’s coal production is used domestically, 15% of which for power generation (RME 2020). These figures are also good indicators for the majority sources of the GHG, hence CO_2_ emissions in the country since a large amount of GHG emissions resulted from natural gas consumption (about 54%), followed by coal and oil with the shares of almost 25% and 20% respectively in the same year. The findings of the study conducted by Gurbanov et al. (2023) affirm the findings of our investigation. According to their analysis, the largest impact on CO_2_ emissions as the main contributor of the GHG emissions is attributed to natural gas, which is followed by coal and oil. In addition, our study’s results are aligned with those of earlier research, such as those conducted by Dong et al. (2019a and 2019b) for countries in Europe and Eurasia, including Russia; and Lotz (2017) for BRICS nations. On the other hand, coal’s contribution to Russia’s energy balance remains currently significant; however, it is expected to decline in near future due to the primary obstacles consisting of diminishing domestic demand for thermal coal, high transportation costs, outdated equipment, congested infrastructure, social unrest in mining districts, and a high rate of coal mine accidents, outlined by recent analyses (Gorbacheva and Sovacool 2015; Lakhno 2015; Pavlov and Petrov 2019). Thus, it is possible to state that these declines may lead to decreasing in related emissions, in line with current study estimates of the GHGE-CCs. As one of the largest GHG emitters of the Africa region, coal is the principal energy source that the energy sector heavily depends on, with a share of roughly 69%, 22%, and 4%, coal, oil, and natural gas in energy consumption of 2023 in South Africa (EI 2024). For many years, South Africa’s economy has relied heavily on coal and the current trends suggest also that this will continue in near future. It is highlighted that a moving away from coal is crucial for South Africa to mitigate the negative effects such as pollution and health from the mining of coal and coal-fired power plants in line with domestic policy and development targets and international agreements to reduce emissions resulting in climate change (Hanto et al. 2022). In order to achieve this target or transition, Hanto et al. (2022) identified four major objectives, affecting the country’s energy sector as (i) accessibility to energy, (ii) keeping the coal industry profitable, (iii) protecting the environment and climate, and (iv) lowering disparities and employment insecurity. However, how rapidly coal will be phased out is still uncertain due to the factors such as growing population, economic expansion, and country’s development leading to increasing the needs for both FFs and electricity. Hence, it can be inferred from the current trends and projections that the GHGE-CCs and other pollutants will exhibit a rising trend in the next years as estimated in the present study. As a country that is not officially a member of the BRICS, Turkiye is a nation that imports energy, with almost over half of its energy needs. The current status of the energy usage of the country makes it clear that the FFs (oil, coal, and natural gas) have been used much more than it does renewable energy sources. The most popular energy source was oil, with 2.30 exajoule in 2023, followed by natural gas (1.74 exajoule) and coal (1.65 exajoule) (EI 2024). Given that the dependence on oil as primary energy source in Turkiye, the government has already stated that its intends to identify more coal mines and expand the existing ones in order to lessen the country’s reliance on foreign sources for energy (Bostanoğlu 2020). Insisting on further increase in coal use in Turkiye may accelerate the increase in GHGE-CCs as it is the largest contributor to anthropogenic GHG emissions that lead to climate change. In addition, it may also cause serious health problems. In this framework, when taking into consideration the country’s national energy strategy, which calls for increasing the proportion of coal in the national energy mix, it can be strongly suggested that Turkiye does not currently have a clear strategy for reducing emissions. This intend may also lead to increases in GHGE-CCs, aligning with the current study estimations.

**Fig. 10:**
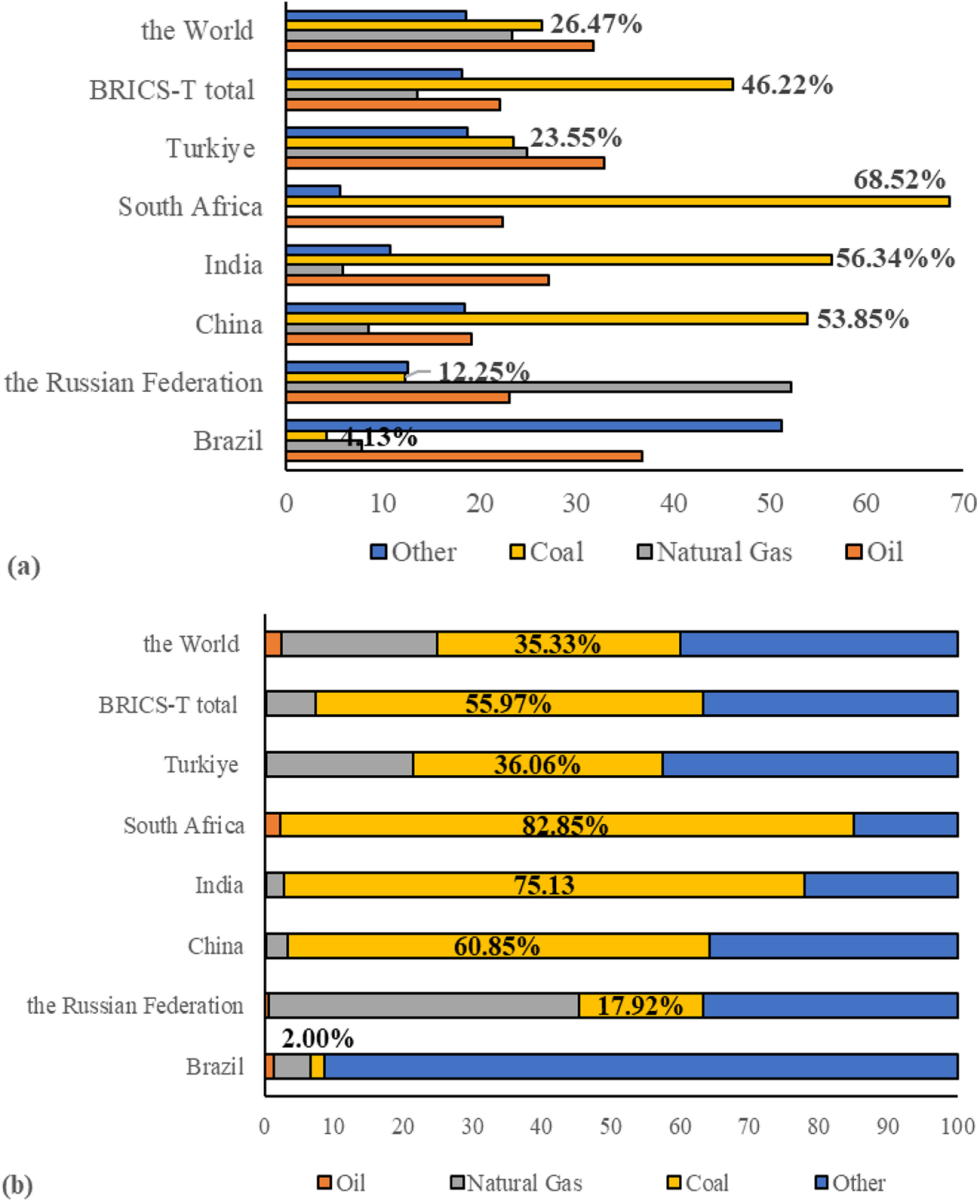
**a** Primary energy consumption by fuel type and **b** shares of the fuel type in power generation in 2023 (other includes the nuclear, hydropower, and renewables) (EI 2024)

It is widely known that coal has a significant impact on the world’s energy supply and GHG emissions, as well as on the energy supply and emissions of the BRICS-T countries. Namely, in 2023, the share of the coal in the world and the BRICS-T total PECs were 26.47% and 46.22%. In the same year, 35.33% of world power generation was supplied by coal, while those for the BRICS-T was 55.97%. The recent data by EI (2024) showed that among these countries, South Africa, India, China, and partly Turkiye rely even more on coal heavily for both PECs and power generation than the Russian Federation and Brazil (Fig. 10a and b). Given the coal-fired power plants produce most of the GHG emissions associated with the burning of coal and the importance of coal in the energy balance of the related nations, attention should be concentrated on the coal use and coal-fired power plants. In this regards, it would be appropriate to promote the adoption of sustainable energy resources and/or implement technologies that can prevent the majority of CO_2_ emissions from large point sources that burn (e.g., coal-fueled power plants) or gasify coal from entering the atmosphere. For instance, despite lack of widespread and commercial availability for coal-fired power plants yet, the CCS technologies could be a good fit since they allow CO_2_ to be separated from other exhaust gasses, compressed for pipeline transportation, and stored underground to prevent it from being released into the atmosphere indefinitely. Of course, investing in large-scale CCS projects may present some challenges. Many of these challenges, meanwhile, can be addressed by government incentives and regulations that are consistent with national climate policy’s aims for reducing GHG emissions. Further and continuous government investment for CCS research, development, and demonstration is also necessary. This approach may result in cutting-edge CCS solutions that are more affordable than those being evaluated for widespread implementation. In addition, GHG emissions can be significantly decreased by increasing the efficiency of coal-fueled power plants as an another substantial mitigation option. In comparison to power plants constructed with the most recent technologies, older and less efficient units will emit more emissions per megawatt-hour of power generation. Therefore, cutting-edge materials intended to facilitate the high-efficiency construction of ultra-supercritical pulverized coal power plants should be the focus of current research and advances in the relevant field or technology. In this case, policies that promote the building of high-efficiency plants are also required. As a result, the amount of GHG emissions from coal-fueled power generation might be reduced in nations that rely significantly on coal, such as China, South Africa, India, and Turkiye, with the implementation of CCS and highly efficient coal-fired power plants.

## Conclusions

This paper, setting out to contribute and improve the existing literature by proposing new and simple models to estimate the GHGE-CCs of the BRICS-T countries, has mainly reached the following conclusions.

i) It was determined that the BRICS-T countries represent more than 42% of the world’s population and govern roughly 27% of global economy. Additionally, it was reported that the BRICS-T countries hold around 8.5%, 25%, and 41% of confirmed reserves of oil, natural gas, and coal in the world, respectively. Besides these fuels, it was recorded that they have also significant nuclear, hydroelectric, and renewable energy resources. Moreover, it was discovered that the BRICS-T countries accounted around 46% and 48% of the world’s greenhouse gas and CO_2_ emissions, respectively.
ii) It was disclosed that the derived models could be used efficiently for estimating the greenhouse gas emissions associated with coal combustion of the BRICS-T countries with accuracy ranges from at least 90% to almost 98%. These results confirm the TA’s usefulness in deriving the estimating models for the greenhouse gas emissions associated coal combustion.
iii) It was estimated that the total greenhouse gas emissions associated with coal combustions in the group and world will increase to 14 BtCO_2eq_ and 19 BtCO_2eq_, with increase rate of around 35% and 34% respectively by 2035, when compared to the data in 2020. Aside the Russian Federation, the estimated results indicated that significant increases are expected for all countries individually in the bloc.

## Limitations of the current study and future work

For the previous four decades, the world has experienced significant economic growth associated with the massive EC. This growth has also brought environmental concerns such as rising the GHG emissions, hence global warming. BRICS-T countries considerably contribute to this growth and concerns as well. Thus, recent studies have concentrated on these rapidly growing and emerging economies. It is evident that this paper concentrated only to BRICS-T countries. Hence, the results do not have the potential for generalization to other countries. Therefore, future studies can spread this paper by adding several variables affecting the GHG emissions directly in the related group for deriving the predictive models. It is strongly recommended that similar studies should be conducted for other regional groups and/or more specifically other countries. Researchers may also conduct the studies in which different modeling techniques are utilized for the group of countries including BRICS-T. Moreover, our study/analysis has certain limitations, which may also be regarded as starting points for additional study on the subject. Firstly, the data used for analysis cover the mostly the emissions from the coal-fired power plants. The missing data, which belong to the other sectors using coal, can make our analysis incomplete. In future, the missing data, coming from almost all sectors, should be explored to construct more comprehensive emission models for the relevant nations. Secondly, it is related to the historical trend of the data since the TA puts out the notion that readers may predict what will happen in the future by looking at what has already happened in the past. Future trends may not present the similarity with past trends. In this case, other variables should be taken into consideration when deriving the predictive models. Therefore, in order to make a generalization of the current results, predictive models for GHGE-CCs should be derived for other computing techniques as well. Thirdly, the current paper is incapable of taking into account a possible decline in group collaboration on climate goals, due to the fact that the data used in this study was from well before February 24, 2022, when Russia began its invasion of Ukraine. How Russian officials will rejoin the climate negotiations is still up in the air. In addition to seriously disrupting climate talks and efforts, tensions surrounding Russia’s invasion of Ukraine may increase the country’s reliance on FFs to offset budgetary deficits and maintain an internationally sound economy. This will require a further study, reassessed and re-scenarized. Although the aforementioned innovative study is anticipated to show a more significant departure from Russian commitments, the current study makes a significant contribution to the body of literature by offering assessments during peacetime.

In conclusion, this paper aims at fulfilling the research gap by proposing simple predictive models for the GHGE-CCs estimation of the BRICS-T countries with the TA. It is thought that the results of this paper shed some light on the importance of the GHG emissions for future studies on climate change policy, associated with fuel combustion and can prove to be beneficial for all the participants.

## Data Availability

Data are provided in this manuscript. There is no supplementary information.

## Nomenclature

3SLS: Three-stage least square method
ARIMA: Autoregressive integrated moving average model
ABC: Artificial bee colony
AI: Artificial intelligence
AMG: Augmented mean group
ANEC: Alternative and nuclear energy consumption
ANN: Artificial neural network
ARDL: Panel autoregressive distributed lag
BPNN: Back propagation EC neural network
BR-AGM (1,1): Adaptive grey model with buffered rolling mechanism
BRICS: Brazil, the Russian Federation, India, China, and South Africa
BRICS-T: Brazil, the Russian Federation, India, China, South Africa—Turkiye
BSA: Backtracking search algorithm
BtCO_2eq_: Billion tonnes of carbon dioxide equivalent
Btoe: Billion tons of oil equivalent
CC: Coal consumption
CCS: Carbon capture and storage
CFCs: Chlorofluorocarbons
CH_4_: Methane
CO_2_: Carbon dioxide
CRWC: Combustible renewables and waste energy consumption
CV: Coefficient of variation
DDF: Improved directional distance function
DGMs: Discrete grey models
DGPM: Discrete grey power model
DL: Deep learning
EC: Energy consumption
EE: Energy efficiency
EEMD-PSOBP: Ensemble empirical mode decomposition-the back propagation neural network based on particle swarm optimization
EG: Electricity generation
EKC: Environmental Kuznets curve
ENGM(1,4): A novel nonlinear multivariate grey model
EO: Equilibrium optimizer
EP-EC: Energy production-consumption
EU: European Union
EU-T: Energy use for transportation
FC: Fuel consumption
FDI: Foreign direct investment
FFs: Fossil fuels
FFC: Fossil fuel consumption
FF-CO_2_: Fossil fuel-related CO_2_ emissions
FT: Freight turnover
GDPs: Gross domestic products
GHG: Greenhouse gas
GHGs: Greenhouse gasses
GHGE-CC: Greenhouse gas emissions associated with coal combustion
HFCs: Hydrofluorocarbons
GM(1,4): Grey model
GMC(1,4): Grey model with convolution integral
GPM: Grey prediction model
GPR: Gaussian processes regression
GWO: Grey wolf optimization
GWP: Global warming potential
HPCT: Heterogeneous panel causality tests
HPEC: Hydropower energy consumption
INFI: Infrastructural investments
JB: Jarque-Bera
LNCT: Updated linear and nonlinear cointegration techniques
LSA: Lightning search algorithm
LSTM: Long-short term memory
M1, M2, M3, M4, M5: Types of the rolling DGPM (1;N) models
MARS: Multivariate adaptive regression splines
MCOLS: Modified completely ordinary least squares
MG: Mean group
MPA: Marine Predators Algorithm
MRIO: A multi-regional input–output model
MtCO_2eq_: Million tonnes of carbon dioxide equivalent
Mtoe: Million tonnes of oil equivalent
NARDL: Non-linear autoregressive distributed lag model
NDHPC: The newly developed hidden panel cointegration
NGC: Natural gas consumption
NMV: Number of motor vehicles
N_2_O: Nitrous oxide
NPADRL: Nonlinear panel autoregressive distributive lag model
NUC: Nuclear energy consumption
O_3_: Ozone
OC: Oil consumption
OGM (1,1): Optimized grey model
PC: Panel cointegration
PEC: Primary energy consumption
PFCs: Perfluorocarbons
PMG: Pool mean group
PM_2.5_ and PM_10_: Particulate matter
PQR: Panel quantile regression
PSO: Particle swarm optimization
PSO-GPR: Particle swarm optimization-Gaussian processes regression
PTM: Panel threshold model
PV: Passenger vehicles
*R*^2^: The coefficient of determination
RA: Regression analysis
RE: Renewable energy
REC: Renewable energy consumption
REP: Renewable energy production
SD: Standard deviation
SGPRT: Second-generation panel unit root test
SF6: Sulfur hexafluoride
SMOSVM: Support vector machine model with sequential minimal optimization
SOS: Symbiotic organisms search
SVM: Support vector machine
SVR: Support vector regression
TA: Trend analysis
TLBO: Teaching–learning-based optimization
TP: Total population
UP: Urban population
UR: Urbanization rate
USA: United States of America
VOCs: Volatile organic compounds
